# Switching to rosuvastatin plus ezetimibe in statin-treated stroke patients with low-density lipoprotein cholesterol levels above 70 mg/dL (SWITCH): a prospective observational study

**DOI:** 10.1186/s12944-025-02781-6

**Published:** 2025-11-12

**Authors:** Wookjin Yang, Yeong-Bae Lee, Eung-Gyu Kim, Han-Jin Cho, Sungwook Yu, Joon-Tae Kim, Jong Wook Shin, Soo Joo Lee, Beom Joon Kim, Ji Man Hong, Seong-Ho Koh, Sang Joon An, A-Hyun Cho, Jin-Man Jung, Hyun-Ji Cho, Chulho Kim, Eung-Joon Lee, Jeong-Min Kim, Seung-Hoon Lee

**Affiliations:** 1 Department of Neurology, Asan Medical Center, Seoul, Korea; 2 Department of Neurology, Gil Medical Center, Gachon University College of Medicine, Incheon, Korea; 3 Department of Neurology, Inje University Busan Paik Hospital, Inje University College of Medicine, Busan, Korea; 4 Department of Neurology, Pusan National University Hospital, Pusan National University School of Medicine, Busan, Korea; 5 Department of Neurology, Korea University Anam Hospital, Korea University College of Medicine, Seoul, Korea; 6 Department of Neurology, Chonnam National University Hospital, Chonnam National University Medical School, Gwangju, Korea; 7 Department of Neurology, Chungnam National University Hospital, Chungnam National University College of Medicine, Daejeon, Korea; 8 Department of Neurology, Daejeon Eulji Medical Center, Eulji University School of Medicine, Daejeon, Korea; 9 Department of Neurology, Seoul National University Bundang Hospital, Seoul National University College of Medicine, Seongnam, Korea; 10 Department of Neurology, Ajou University Medical Center, Ajou University School of Medicine, Suwon, Korea; 11 Department of Neurology, Hanyang University Guri Hospital, Hanyang University College of Medicine, Guri, Korea; 12 Department of Neurology, International St. Mary’s Hospital, Catholic Kwandong University College of Medicine, Incheon, Korea; 13 Department of Neurology, Yeouido St. Mary’s Hospital, The Catholic University of Korea College of Medicine, Seoul, Korea; 14 Department of Neurology, Korea University Ansan Hospital, Korea University College of Medicine, Ansan, Korea; 15 Department of Neurology, Incheon St. Mary’s Hospital, The Catholic University of Korea College of Medicine, Incheon, Korea; 16 Department of Neurology, Hallym University Chuncheon Sacred Heart Hospital, Hallym University College of Medicine, Chuncheon, Korea; 17 Department of Neurology, Seoul National University Hospital, Seoul National University College of Medicine, 101 Daehak-ro, Jongno-gu, Seoul, 03080 Korea

**Keywords:** Ezetimibe, Hydroxymethylglutaryl-CoA reductase inhibitors, Low-density lipoprotein cholesterol, Stroke

## Abstract

**Background:**

Effective lipid management is critical for secondary stroke prevention, however, many patients fail to achieve target low-density lipoprotein cholesterol (LDL-C) levels with statin monotherapy. This study evaluated the real-world effectiveness and safety of switching from statin monotherapy to rosuvastatin plus ezetimibe combination therapy (REZ) in patients with stroke.

**Methods:**

This multicenter, prospective, observational study enrolled patients with stroke and baseline LDL-C ≥ 70 mg/dL despite statin monotherapy from 16 Korean stroke centers. Participants were switched to REZ at doses of 5/10 mg, 10/10 mg, or 20/10 mg at the investigators’ discretion. Lipid profiles were assessed at three and six months. The primary outcome was achieving LDL-C **<** 70 mg/dL at six months.

**Results:**

In total, 1,431 participants enrolled between May 2021 and March 2023 were eligible (mean age 65.3 ± 10.6 years; 66.8% male). Among 994 participants completing follow-up, the mean baseline LDL-C was 98.9 ± 22.4 mg/dL. At six months, 708 (71.2%) achieved LDL-C < 70 mg/dL. Mean LDL-C decreased to 62.7 ± 22.1 mg/dL at three months and to 62.0 ± 22.0 mg/dL at six months. The effectiveness of REZ remained consistent across different REZ dosages and regardless of changes in statin intensity during the switch. REZ was particularly effective in patients with diabetes (odds ratio [95% confidence interval], 1.85 [1.32–2.59]; *P* < 0.001) and baseline LDL-C 70–99 mg/dL (2.71 [2.04–3.59]; *P* < 0.001). Fewer participants achieved stricter targets (LDL-C < 55 mg/dL or LDL-C < 70 mg/dL plus 50% reduction).

**Conclusions:**

Switching to REZ effectively reduced LDL-C in patients with stroke receiving statin monotherapy with LDL-C ≥ 70 mg/dL, offering potential benefits for secondary cardiovascular prevention in real-world practice.

**Supplementary Information:**

The online version contains supplementary material available at 10.1186/s12944-025-02781-6.

## Introduction

Patients with stroke are at high risk of recurrent stroke and other cardiovascular diseases (CVDs), necessitating effective secondary prevention strategies. Lipid-lowering therapy is a cornerstone of mitigating recurrent CVD risk, with accumulating evidence supporting progressively lower low-density lipoprotein cholesterol (LDL-C) targets [1–7], summarized by the widely recognized notion that “the lower, the better”. Current stroke guidelines also recommend high-intensity statin for non-cardioembolic stroke and advocate achieving LDL-C levels below 70 mg/dL in patients with atherosclerotic disease, based on the SPARCL and TST trials [8–10]. Given the high prevalence of coexisting atherosclerotic disease among patients with stroke [11, 12], achieving stringent LDL-C targets below 70 mg/dL is essential for effective secondary prevention.

Statins are the first-line agents for lipid-lowering therapy and are widely prescribed. However, many patients, particularly those with ischemic stroke, face challenges managing cholesterol with statin monotherapy due to higher cholesterol levels and significant atherosclerotic burden, necessitating additional lipid-lowering agents [13, 14]. In such cases, the combination of statin and ezetimibe, demonstrated by the IMPROVE-IT study to significantly enhance LDL-C reduction and cardiovascular outcomes [3], has become a widely used strategy in clinical practice. Nevertheless, real-world data on the effectiveness and safety of this combination, especially among patients with stroke in Korea, remains limited.

Thus, this study aimed to evaluate the effectiveness and safety of switching to a rosuvastatin plus ezetimibe combination therapy (REZ) in Korean patients with stroke who were already on statins but did not achieve an LDL-C level of < 70 mg/dL in routine clinical practice. It was hypothesized that switching to REZ would lead to meaningful LDL-C reduction without compromising safety.

## Methods

### Study design and population

This prospective, observational, non-interventional, single-group study was conducted at 16 Korean stroke centers, with patient enrollment between May 2021 and March 2023. Adult participants aged ≥ 19 years with a history of stroke and an LDL-C level ≥ 70 mg/dL despite statin monotherapy were eligible. Exclusion criteria were: (1) hypersensitivity to rosuvastatin or ezetimibe; (2) active liver disease; (3) muscular disorders; (4) concomitant cyclosporin use; (5) severe renal disease whose creatinine clearance < 30 mL/min; (6) pregnancy or breastfeeding; and (7) other conditions deemed unsuitable by investigators. Following the baseline lipid profile assessment, eligible participants were switched from statin monotherapy to REZ. The dose of REZ was determined at the physicians’ discretion, and lipid profiles were monitored three and six months after the switch. This study was approved by the institutional review board (IRB) of Seoul National University Hospital (IRB No. H-2103-061−1204) and the IRBs of all participating sites. Written informed consent was obtained from all participants or their caregivers/guardians.

### Clinical information

For all participants, data on age, sex, height, weight, comorbidities (hypertension, diabetes, dyslipidemia, atrial fibrillation, and coronary artery disease), smoking status, time since stroke diagnosis, stroke subtype, duration of prior statin monotherapy, and the type and dose of statin used at baseline were collected and recorded in electronic case report forms. Body mass index was calculated based on height and weight. Statin intensity was categorized as high (atorvastatin 40–80 mg, rosuvastatin 20–40 mg), moderate (atorvastatin 10–20 mg, rosuvastatin 5–10 mg, pitavastatin 1–4 mg, simvastatin 20–40 mg, pravastatin 40–80 mg, lovastatin 40–80 mg, fluvastatin 80 mg), and low (simvastatin 10 mg, pravastatin 10–20 mg, lovastatin 20 mg, fluvastatin 20–40 mg) [15]. Change in statin intensity was defined as maintained (e.g., a moderate-intensity statin to moderate-intensity REZ), reduced (e.g., a high-intensity statin to moderate-intensity REZ), or escalated (e.g., a moderate-intensity statin to high-intensity REZ) based on adjustments made during the switch to REZ.

### Study outcomes

The primary effectiveness outcome was the proportion of participants achieving LDL-C < 70 mg/dL at six months after switching to REZ. Secondary effectiveness outcomes included: (1) the proportion of participants achieving both LDL-C < 70 mg/dL and ≥ 50% reduction from baseline at six months; (2) the proportion achieving LDL-C < 55 mg/dL at six months; and (3) changes and percent changes in the lipid profiles (i.e., LDL-C, high-density lipoprotein cholesterol [HDL-C], total cholesterol, non-HDL-C, and triglyceride) at three and six months. Safety outcomes encompassed total and unexpected adverse events, adverse drug reactions, serious adverse events, and serious adverse drug reactions during the six-month follow-up, coded using the Medical Dictionary for Regulatory Activities (v26.1).

### Statistical analysis

Effectiveness outcomes were assessed in the full analysis set, comprising participants with both baseline and six-month LDL-C data. Safety outcomes were evaluated in the safety set, including all participants who received at least one dose of REZ during the study period. Continuous variables were reported as mean ± standard deviation, and categorical variables as numbers and/or percentages. Proportions of participants achieving LDL-C targets were expressed as percentages with 95% confidence intervals. Changes and percent changes of lipid profiles from baseline were analyzed with paired t-tests. Logistic regression was conducted to identify patient characteristics associated with the odds of achieving LDL-C < 70 mg/dL: age, sex, body mass index, comorbidities, smoking status, prior statin intensity and type, baseline LDL-C levels, REZ dose, and change in statin intensity during the switch to REZ. Statistical significance was determined as a two-sided probability value of < 0.05. Statistical analyses were performed using SAS (v9.4, SAS Institute Inc., Cary, NC, USA) and R (v4.4.2, R Foundation, Vienna, Austria).

## Results

**Fig. 1:**
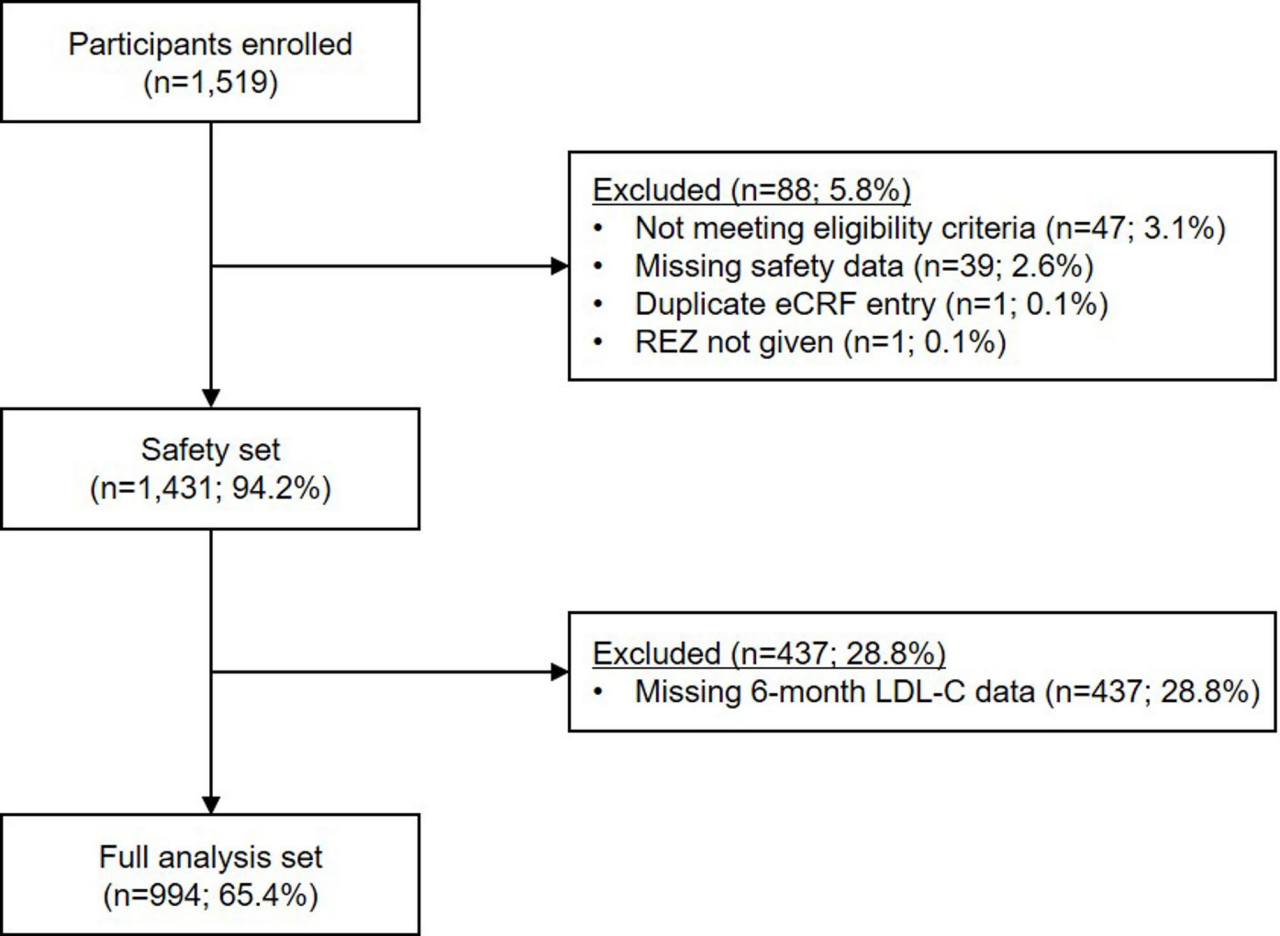
Study flowchart. eCRF, electronic case report form; LDL-C, low-density lipoprotein cholesterol; REZ, rosuvastatin plus ezetimibe combination therapy

Among the 1,519 electronic case report forms collected, 1,431 participants met the eligibility criteria and received at least one dose of REZ during the study period, comprising the safety set. Of these, 994 participants with available LDL-C levels at six months were included in the full analysis set (Fig. 1). Baseline characteristics were summarized in the safety set. The participants had a mean age of 65.3 ± 10.6 years, with 66.8% (*n* = 956) being men. Nearly all participants experienced an ischemic stroke (*n* = 1,424; 99.5%), with a mean time since diagnosis of 59.5 ± 60.1 months before enrollment. Atorvastatin (*n* = 885; 61.8%) was the most commonly used statin, with moderate-intensity statins being the most frequent in terms of intensity (*n* = 995; 69.5%). Further details are provided in Table 1 and Table S1.

**Table 1:** Baseline characteristics of the study population

Variables	Total (*n* = 1,431)
Age, years	65.3 ± 10.6
Male sex	956 (66.8%)
Body mass index, kg/m^2^	24.8 ± 3.3
Hypertension	953 (66.6%)
Diabetes	357 (25.0%)
Dyslipidemia	373 (26.1%)
Atrial fibrillation	84 (5.9%)
Coronary artery disease	62 (4.3%)
Ever smoking^a^	243 (18.8%)
Time since stroke diagnosis, months	59.5 ± 60.1
Stroke subtype	
Ischemic stroke	1,424 (99.5%)
Hemorrhagic stroke	7 (0.5%)
Duration of prior statin monotherapy, months^b^	28.2 ± 35.0
Previous statin	
Atorvastatin	885 (61.8%)
Rosuvastatin	376 (26.3%)
Pitavastatin	127 (8.9%)
Others	43 (3.0%)
Previous statin intensity	
Low-intensity	22 (1.5%)
Moderate-intensity	995 (69.5%)
High-intensity	414 (28.9%)

Data are presented as number (%) or mean ± standard deviation

^a^ *n* = 1,290

^b^ *n* = 1,427

**Fig. 2:**
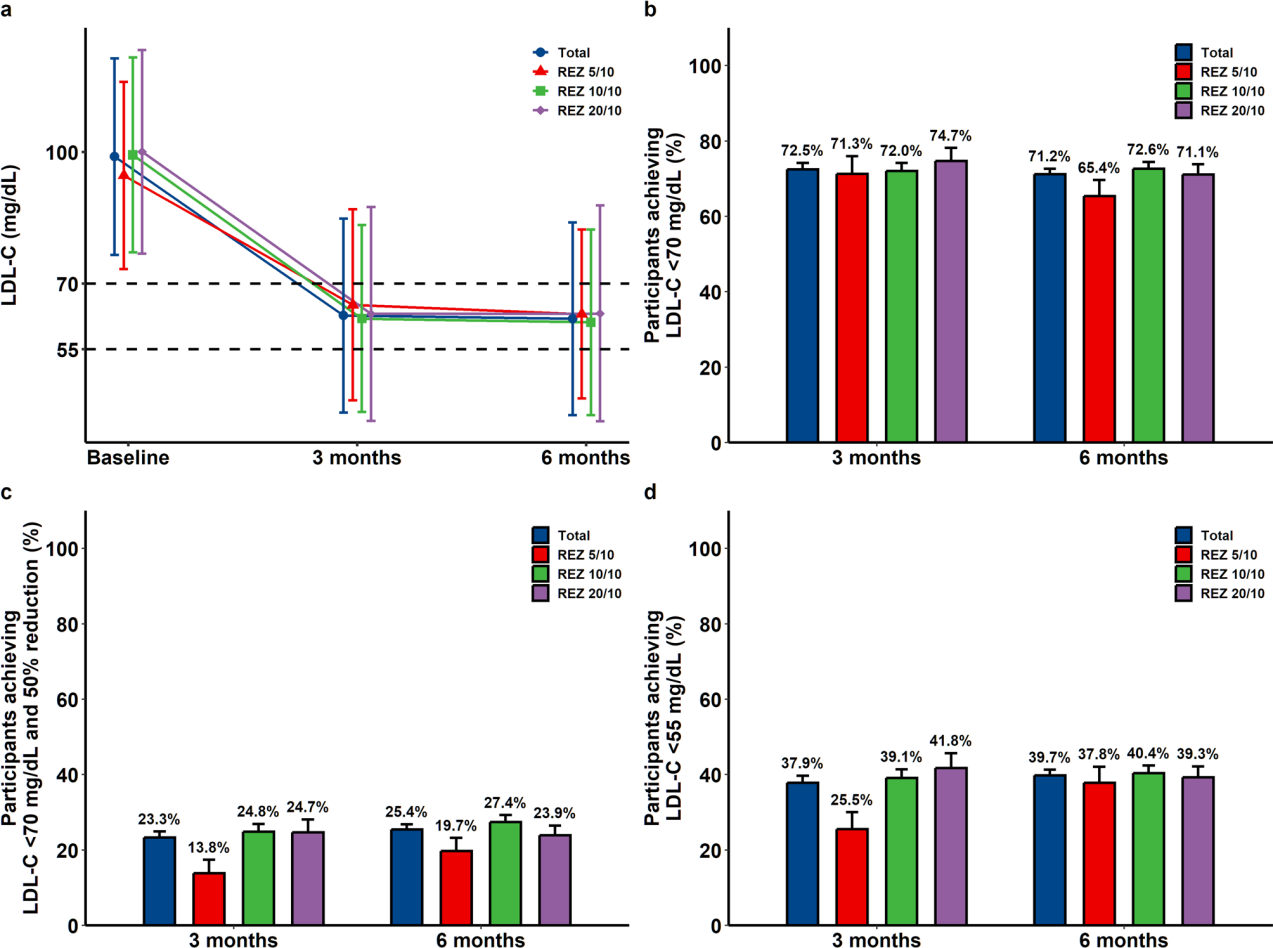
Effectiveness of switching to rosuvastatin plus ezetimibe combination therapy by dose in stroke patients on statin monotherapy. **a** LDL-C change, **b** participants achieving LDL-C < 70 mg/dL, **c** participants achieving LDL-C < 70 mg/dL with a reduction ≥ 50%, and (**d**) participants achieving LDL-C < 55 mg/dL, all assessed at three and six months. Data are presented as mean values with standard deviations (**a**) or proportions with standard errors (**b**–**d**). LDL-C, low-density lipoprotein cholesterol; REZ, rosuvastatin plus ezetimibe combination therapy

**Fig. 3:**
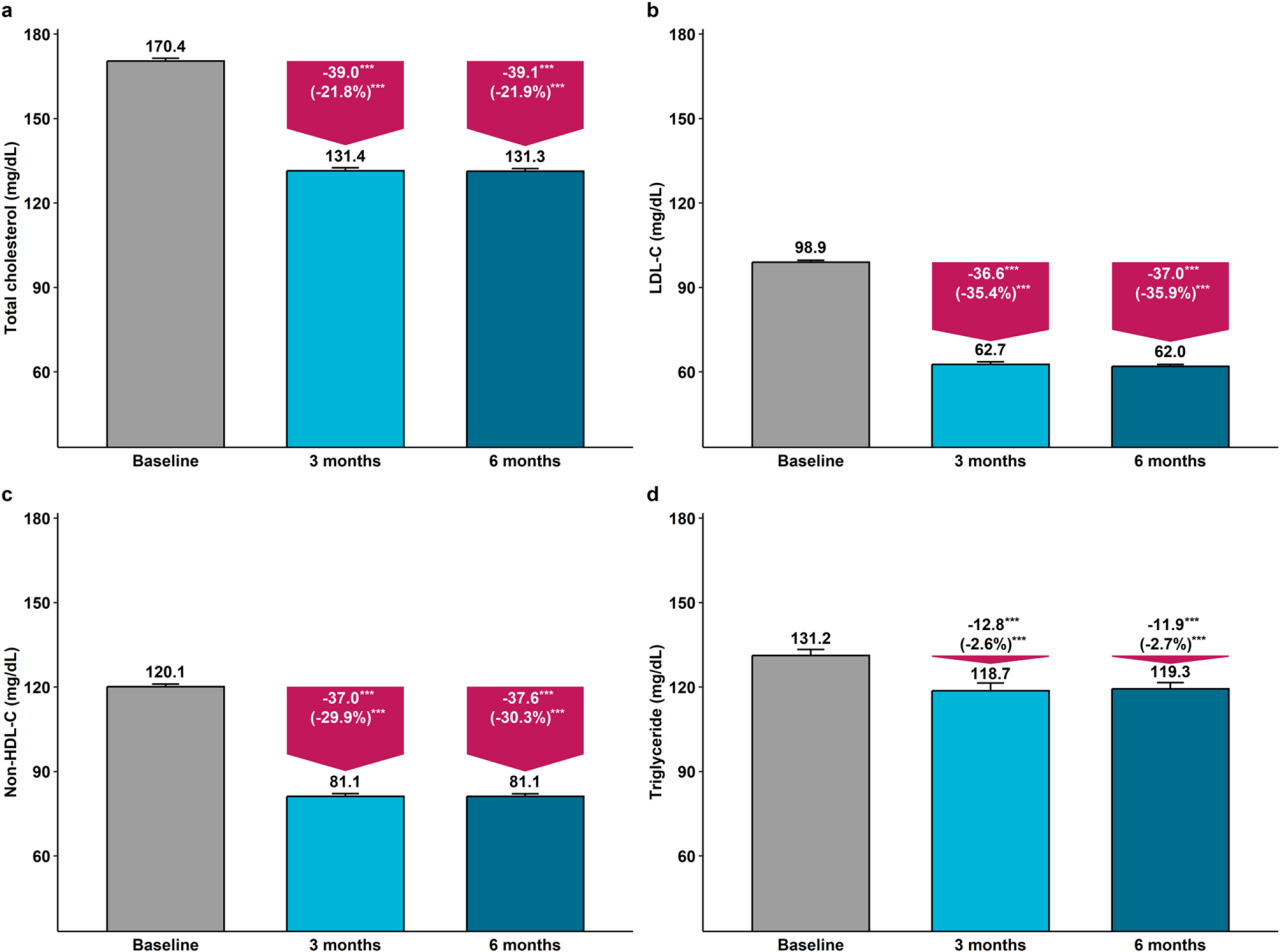
Changes in lipid profiles after switching to rosuvastatin plus ezetimibe combination therapy in stroke patients on statin monotherapy. Panels illustrate (**a**) total cholesterol, **b** LDL-C, **c** non-HDL-C, and **d** triglyceride. Bar graphs show mean lipid concentrations at baseline, 3 months, and 6 months, with error bars indicating standard errors. Downward pink arrows highlight absolute and percent changes from baseline at each follow-up. ^***^P < 0.001 compared with baseline. HDL-C, high-density lipoprotein cholesterol; LDL-C, low-density lipoprotein cholesterol

Effectiveness outcomes were assessed in the full analysis set. REZ was administered at doses of 5/10, 10/10, and 20/10 mg to 127 (12.8%), 587 (59.1%), and 280 (28.2%) participants, respectively. Switching from statin monotherapy to REZ reduced the mean LDL-C level from 98.9 ± 22.4 mg/dL at baseline to 62.7 ± 22.1 mg/dL at three months and to 62.0 ± 22.0 mg/dL at six months. At six months, 708 (71.2%) participants achieved LDL-C < 70 mg/dL, the primary effectiveness outcome. Additionally, 253 (25.5%) achieved both LDL-C < 70 mg/dL and a 50% reduction from baseline, while 395 (39.7%) reached LDL-C < 55 mg/dL with REZ treatment (Table 2). No clear dose-response relationship of REZ was observed in patients with stroke treated with statins (Fig. 2). Other lipid profiles, including total cholesterol, non-HDL cholesterol, and triglyceride, also showed significant reduction after switching to REZ (Fig. 3).

**Table 2:** Effectiveness of switching to rosuvastatin plus ezetimibe combination therapy in stroke patients on statin monotherapy

	Baseline	3 months	6 months
LDL-C level, mg/dL (mean ± standard deviation)	98.9 ± 22.4	62.7 ± 22.1	62.0 ± 22.0
LDL-C < 70 mg/dL, n (%; 95% CI)	-	498 (72.5%; 69.0–75.8%)	708 (71.2%; 68.3–74.0%)
LDL-C < 70 mg/dL and 50% reduction, n (%; 95% CI)	-	160 (23.3%; 20.2–26.6%)	253 (25.5%; 22.8–28.3%)
LDL-C < 55 mg/dL, n (%; 95% CI)	-	260 (37.9%; 34.2–41.6%)	395 (39.7%; 36.7–42.9%)

*CI* confidence interval, *LDL-C* low-density lipoprotein cholesterol

**Fig. 4:**
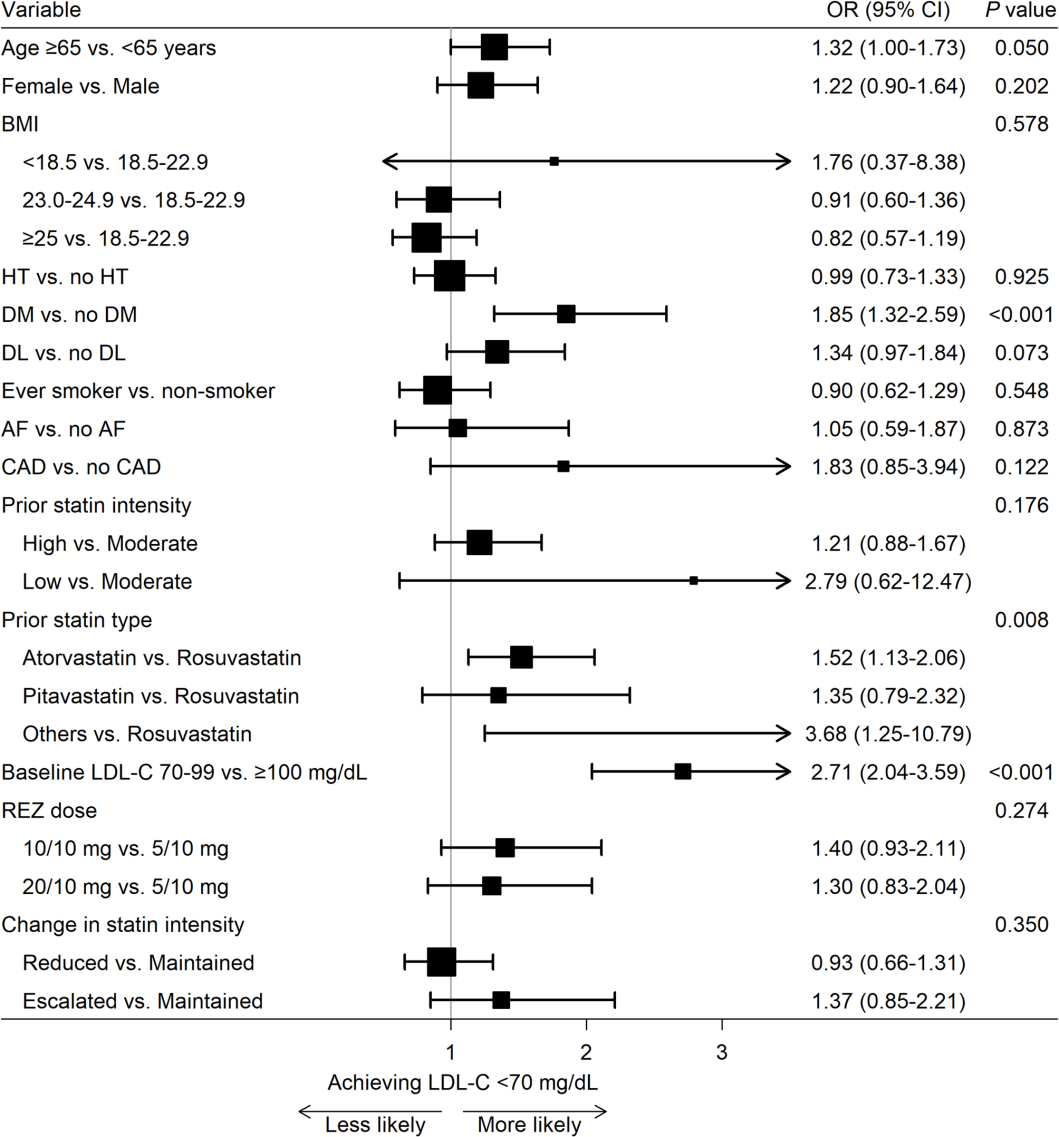
Effectiveness of switching to rosuvastatin plus ezetimibe combination therapy by clinical parameters in stroke patients on statin monotherapy. Forest plot describing the odds ratios (ORs) with 95% confidence intervals (CIs) for the association between the switch to rosuvastatin plus ezetimibe combination therapy and achieving LDL-C level < 70 mg/dL at six months. ORs were calculated from logistic regression analysis and plotted on the x-axis with a log scale, with OR > 1 indicating a greater likelihood of achieving the target. AF, atrial fibrillation; BMI, body mass index; CAD, coronary artery disease; CI, confidence interval; DL, dyslipidemia; DM, diabetes mellitus; HT, hypertension; LDL-C, low-density lipoprotein cholesterol; OR, odds ratio; REZ, rosuvastatin plus ezetimibe combination therapy

Logistic regression showed that participants with diabetes or lower baseline LDL-C levels were more likely to achieve target LDL-C levels at six months. Those previously on atorvastatin or other statins (simvastatin, pravastatin, lovastatin, or fluvastatin) had higher odds of achieving target LDL-C levels compared to those previously on rosuvastatin. Statin intensity was maintained in the majority of participants (*n* = 699; 70.3%) during the switch to REZ, while it was reduced in 196 (19.7%) and escalated in 99 (10.0%). Consistent LDL-C reductions were observed across all three groups (Fig. 4).

In the safety set, a total of 159 adverse events were reported in 124 (8.7%) participants, including 54 adverse drug reactions in 38 (2.7%) participants. Serious adverse events occurred in 28 (2.0%) participants; however, no serious adverse drug reactions were reported. The most common adverse events were dizziness, alanine aminotransferase elevation, and headache. Dizziness, the most frequent adverse drug reaction, occurred at a low rate of 0.35%. Muscle-related adverse events, a key concern with high-intensity statin therapy, were also uncommon in this study (Table 3). Detailed safety outcomes by system organ class are presented in Table S2.

**Table 3:** Most frequently reported adverse events and adverse drug reactions after switching to rosuvastatin plus ezetimibe in statin-treated stroke patients

	Adverse events		Adverse drug reactions	
	Patients, *n* (%)	Events, *n*	Patients, *n* (%)	Events, *n*
Dizziness	15 (1.05%)	15	5 (0.35%)	5
Myalgia	4 (0.28%)	4	4 (0.28%)	4
ALT elevation	8 (0.56%)	8	4 (0.28%)	4
Anesthesia	4 (0.28%)	4	4 (0.28%)	4
Rash	4 (0.28%)	4	2 (0.14%)	2
Muscular weakness	2 (0.14%)	2	2 (0.14%)	2
Pain in extremity	3 (0.21%)	3	2 (0.14%)	2
Nausea	2 (0.14%)	2	2 (0.14%)	2
Facial edema	2 (0.14%)	2	2 (0.14%)	2
Peripheral edema	3 (0.21%)	3	2 (0.14%)	2
Fatigue	3 (0.21%)	3	2 (0.14%)	2
Headache	7 (0.49%)	7	2 (0.14%)	2

*ALT* alanine aminotransferase

## Discussion

This study demonstrated that switching from statin monotherapy to REZ effectively achieved additional LDL-C reduction in patients with stroke. This effect was consistently observed across all REZ doses and was observed regardless of whether statin intensity was maintained, reduced, or escalated during the switch. Switching to REZ was safe, with no serious adverse drug reactions and only a few muscle-related adverse events reported.

Statins have long been the primary therapy for lipid lowering in ischemic stroke, yet fewer than 30% of patients achieve stringent LDL-C targets of < 70 mg/dL in real-world data [13]. In the present study, over 1,400 patients who had received a mean duration of 28.2 months of statin monotherapy—well beyond the period required to reach maximal LDL-C lowering effect [16]—still had LDL-C levels that remained above 70 mg/dL, underscoring the limitations of statin monotherapy. Proprotein convertase subtilisin/kexin type 9 (PCSK9) inhibitors have emerged as potent lipid-lowering agents [6, 7], but their high cost and the inconvenience of subcutaneous administration hinder accessibility and routine use [17]. Consequently, ezetimibe has become the most widely adopted adjunctive therapy in combination with statins. Recent trials further validated the efficacy of moderate-intensity statin plus ezetimibe as an alternative to high-intensity statins in atherosclerotic CVD and ischemic stroke [18, 19], supporting the growing use of combination therapy for achieving stricter LDL-C targets. This study demonstrated that switching to REZ, across all three prescribed doses, allowed approximately 70% of patients with stroke and insufficient response to statin monotherapy to achieve target LDL-C levels below 70 mg/dL in real-world practice. The proportion achieving both LDL-C < 70 mg/dL and a ≥ 50% reduction was lower, likely because participants had only moderately elevated baseline LDL-C levels after prior statin use and therefore had limited scope for an additional ≥ 50% reduction rather than reflecting limited effectiveness. Additionally, no clear dose–response relationship for REZ effectiveness was observed in this study. A possible explanation is that most patients were already receiving moderate- or high-intensity statins, where the additional lipid-lowering effect of the ezetimibe combination likely outweighed any incremental benefit of statin dose escalation. The lack of significant differences in lipid-lowering effect, regardless of whether prior statin intensity was maintained or changed during the switch to REZ, may further support this interpretation. Considering the tolerability of REZ, including the low rate of muscle-related adverse events, this study suggests that combining ezetimibe with statins may be preferable to escalating statin intensity for achieving target LDL-C levels in patients with stroke unresponsive to statin monotherapy.

Patients with diabetes appeared to benefit more from switching to REZ compared with those without diabetes. This interesting finding aligns with previous studies showing greater cardiovascular benefits of ezetimibe combination in diabetic populations [20–22]. This enhanced benefit is suggested to be due to increased expression of Niemann-Pick C1-like 1, a direct target of ezetimibe [23–25]. The present study, along with prior evidence, suggests that ezetimibe combination could be prioritized in patients with diabetes as well as stroke. Differences in LDL-C target achievement rates were observed based on the type of prior statin, with prior atorvastatin users more frequently achieving LDL-C < 70 mg/dL than rosuvastatin users. Although both are moderate- to high-intensity statins, rosuvastatin has been reported to have a slightly stronger lipid-lowering effect than atorvastatin [26]. Switching from atorvastatin to REZ may allow more room for further statin escalation, enabling greater LDL-C reductions than switching from rosuvastatin. Nonetheless, despite this heterogeneity, approximately 65% of patients previously on rosuvastatin achieved target LDL-C levels, indicating that switching to REZ is an effective therapeutic option for these patients as well.

### Strengths and limitations

A key strength of this study lies in providing practical data on the effectiveness and safety of switching to ezetimibe combination therapy in Korean patients with stroke and suboptimal responses to statin monotherapy, supported by large sample size. However, several limitations should also be acknowledged. First, as an open-label, observational, single-group study, potential residual confounders affecting lipid-lowering effects (e.g., drug compliance, diet, and exercise) may not have been adequately controlled. Therefore, the direct contribution of the therapy switch to LDL-C reduction cannot be fully ascertained. Second, given the relatively high drop-out rate, the results should be interpreted with caution owing to potential bias arising from participant attrition. Third, the primary outcome of this study was the LDL-C target achievement rate, which does not reflect clinical endpoints such as cardiovascular events. Moreover, despite significant LDL-C reduction with the switch to REZ, the modest attainment of stricter LDL-C targets suggests that this study may provide limited information for high-risk patients who may require additional lipid-lowering therapy (e.g., PCSK9 inhibitors) to achieve guideline-recommended goals. Finally, this study was conducted exclusively in Korean patients, limiting the generalizability of the findings to other ethnicities. Further studies in more diverse populations are warranted.

## Conclusions

In conclusion, switching to REZ was an effective and safe second-line option for patients with stroke who did not achieve target LDL-C levels with statin monotherapy in real-world practice. These findings may provide practical guidance for stroke physicians in optimizing lipid-lowering strategies beyond statin monotherapy, thereby contributing to reducing recurrent CVD.

## Supplementary Information

Supplementary Material 1.

## Data Availability

The datasets used and/or analyzed during the current study are available from the corresponding author on reasonable request.

## Abbreviations

CVD: Cardiovascular disease
HDL-C: High-density lipoprotein cholesterol
IRB: Institutional review board
LDL-C: Low-density lipoprotein cholesterol
PCSK9: Proprotein convertase subtilisin/kexin type 9
REZ: Rosuvastatin plus ezetimibe combination therapy
