# Supplementary material for: Switching to rosuvastatin plus ezetimibe in statin-treated stroke patients with low-density lipoprotein cholesterol levels above 70 mg/dL (SWITCH): a prospective observational study
Source: Lipids Health Dis. 2025 Nov 12;24:359. doi: 10.1186/s12944-025-02781-6 (PMC12613543; doi:10.1186/s12944-025-02781-6)
Supplement: Supplementary file 1 — Supplementary Material 1. [file 12944_2025_2781_MOESM1_ESM.docx]

**Table S1. Baseline characteristics of the full analysis set**

| **Variables** | **Total (n=994)** |
| --- | --- |
| Age, years | 65.6 ± 10.3 |
| Male sex | 676 (68.0%) |
| Body mass index, kg/m^2^ | 24.8 ± 3.2 |
| Hypertension | 686 (69.0%) |
| Diabetes | 267 (26.9%) |
| Dyslipidemia | 269 (27.1%) |
| Atrial fibrillation | 61 (4.3%) |
| Coronary artery disease | 45 (3.1%) |
| Ever smoking^a^ | 173 (19.5%) |
| Time since stroke diagnosis, months | 61.4 ± 60.6 |
| Stroke subtype |  |
| Ischemic stroke | 987 (99.3%) |
| Hemorrhagic stroke | 7 (0.7%) |
| Duration of prior statin monotherapy, months^b^ | 29.4 ± 36.1 |
| Previous statin |  |
| Atorvastatin | 588 (59.2%) |
| Rosuvastatin | 295 (29.7%) |
| Pitavastatin | 80 (8.1%) |
| Others | 31 (3.1%) |
| Previous statin intensity |  |
| Low-intensity | 15 (1.5%) |
| Moderate-intensity | 719 (72.3%) |
| High-intensity | 260 (26.2%) |

Data are presented as number (%) or mean ± standard deviation.

^a^ n=886

^b^ n=993

**Table S2. Full list of safety outcomes categorized by system organ class after switching to rosuvastatin plus ezetimibe**

|  | **Adverse events** | | **Adverse drug reactions** | | **Serious adverse events** | |
| --- | --- | --- | --- | --- | --- | --- |
|  | **Number of patients (%)** | **Number of events** | **Number of patients (%)** | **Number of events** | **Number of patients (%)** | **Number of events** |
| Nervous system disorders | 40 (2.8%) | 41 | 8 (0.6%) | 8 | 11 (0.8%) | 11 |
| General disorders and administration site conditions | 18 (1.3%) | 21 | 9 (0.6%) | 12 | 3 (0.2%) | 3 |
| Investigations | 11 (0.8%) | 18 | 5 (0.4%) | 7 | 0 (0.0%) | 0 |
| Gastrointestinal disorders | 16 (1.1%) | 16 | 8 (0.6%) | 8 | 2 (0.1%) | 2 |
| Musculoskeletal and connective tissue disorders | 12 (0.8%) | 14 | 9 (0.6%) | 11 | 0 (0.0%) | 0 |
| Skin and subcutaneous tissue disorders | 7 (0.5%) | 8 | 4 (0.3%) | 4 | 0 (0.0%) | 0 |
| Injury, poisoning, and procedural complications | 7 (0.5%) | 7 | 0 (0.0%) | 0 | 4 (0.3%) | 4 |
| Metabolism and nutrition disorders | 7 (0.5%) | 7 | 0 (0.0%) | 0 | 0 (0.0%) | 0 |
| Vascular disorders | 7 (0.5%) | 7 | 1 (0.1%) | 1 | 1 (0.1%) | 1 |
| Infections and infestations | 6 (0.4%) | 6 | 0 (0.0%) | 0 | 2 (0.1%) | 2 |
| Neoplasms benign, malignant, and unspecified | 4 (0.3%) | 4 | 0 (0.0%) | 0 | 4 (0.3%) | 4 |
| Cardiac disorders | 3 (0.2%) | 3 | 1 (0.1%) | 1 | 1 (0.1%) | 1 |
| Respiratory, thoracic, and mediastinal disorders | 2 (0.1%) | 2 | 0 (0.0%) | 0 | 1 (0.1%) | 1 |
| Blood and lymphatic system disorders | 1 (0.1%) | 1 | 0 (0.0%) | 0 | 0 (0.0%) | 0 |
| Eye disorders | 1 (0.1%) | 1 | 0 (0.0%) | 0 | 0 (0.0%) | 0 |
| Hepatobiliary disorders | 1 (0.1%) | 1 | 1 (0.1%) | 1 | 0 (0.0%) | 0 |
| Renal and urinary disorders | 1 (0.1%) | 1 | 1 (0.1%) | 1 | 0 (0.0%) | 0 |
| Reproductive system and breast disorders | 1 (0.1%) | 1 | 0 (0.0%) | 0 | 0 (0.0%) | 0 |
| Total | 124 (8.7%) | 159 | 38 (2.7%) | 54 | 28 (1.9%) | 29 |
